# Patterns of Cytogenomic Findings from a Case Series of Recurrent Pregnancy Loss Provide Insight into the Extent of Genetic Defects Causing Miscarriages

**DOI:** 10.1055/s-0044-1785227

**Published:** 2024-03-29

**Authors:** Autumn DiAdamo, Hongyan Chai, Mei Ling Chong, Guilin Wang, Jiadi Wen, Yong-Hui Jiang, Peining Li

**Affiliations:** 1 Department of Genetics, Yale University School of Medicine, New Haven, Connecticut, United States; 2 Yale Center for Genome Analysis, New Haven, Connecticut, United States; 3 Yale Center for Genomic Health, Yale University School of Medicine, New Haven, Connecticut, United States

**Keywords:** recurrent pregnancy loss, products of conception, chromosomal abnormalities, pathogenic copy number variants

## Abstract

**Background** A retrospective study was performed to evaluate the patterns of cytogenomic findings detected from a case series of products of conception (POC) in recurrent pregnancy loss (RPL) over a 16-year period from 2007 to 2023.

**Results** This case series of RPL was divided into a single analysis (SA) group of 266 women and a consecutive analysis (CA) group of 225 women with two to three miscarriages analyzed. Of the 269 POC from the SA group and the 469 POC from the CA group, a spectrum of cytogenomic abnormalities of simple aneuploidies, compound aneuploidies, polyploidies, and structural rearrangements/pathogenic copy number variants (pCNVs) were detected in 109 (41%) and 160 cases (34%), five (2%) and 11 cases (2%), 35 (13%) and 36 cases (8%), and 10 (4%) and 19 cases (4%), respectively. Patterns with recurrent normal karyotypes, alternating normal and abnormal karyotypes, and recurrent abnormal karyotypes were detected in 74 (33%), 71 (32%), and 80 (35%) of consecutive miscarriages, respectively. Repeat aneuploidies of monosomy X and trisomy 16, triploidy, and tetraploidy were detected in nine women.

**Conclusions** A comparable spectrum of cytogenomic abnormalities was noted in the SA and CA groups of RPL. A skewed likelihood of 2/3 for recurrent normal and abnormal karyotypes and 1/3 for alternating normal and abnormal karyotypes in consecutive miscarriages was observed. Routine cytogenetic analysis should be performed for consecutive miscarriages. Further genomic sequencing to search for detrimental and embryonic lethal variants causing miscarriages and pathogenic variants inducing aneuploidies and polyploidies should be considered for RPL with recurrent normal and abnormal karyotypes.

## Introduction

Pregnancy loss occurs in 10 to 30% of clinically recognized pregnancies, and approximately 15% of couples undergo a clinically recognized spontaneous pregnancy loss (SPL). 1 Most couples go on to achieve a successful pregnancy after an SPL, but 3 to 5% of couples experience recurrent pregnancy loss (RPL). 2 Earlier data and current cytogenetic analysis detect chromosomal abnormalities, including numerical abnormalities of autosomal trisomies, monosomy X, and polyploidies, as well as structural abnormalities of various types of chromosomal imbalances, in approximately 50% of products of conception (POC). 3 4 5 The analytical validation and clinical utilization of chromosome microarray analysis (CMA) enabled the further characterization of genomic imbalances from structural rearrangements and the detection of pathogenic copy number variants (pCNVs) from POC specimens with culture failure and normal karyotypes. 5 6 7 8

RPL is defined as two or more pregnancy losses according to the guidelines of the American Society for Reproductive Medicine and the European Society of Human Reproduction and Embryology. 9 10 Since chromosomal abnormalities represent the most common cause of pregnancy loss, a detailed analysis of the occurrence and patterns of chromosomal abnormalities in RPL could provide insight into genetic predisposition to meiotic nondisjunction and genomic instability for underlying molecular mechanisms causing miscarriages. 2 3 Cytogenetic analyses on case series of RPL and a recent meta-analysis indicated no difference in the distribution of chromosomal abnormalities between SPL and RPL but probably a significantly higher incidence of chromosomal abnormalities in SPL than in RPL. 11 12 13 14 We performed a retrospective study to evaluate the diagnostic cytogenomic findings from a single analysis (SA) group and a consecutive analysis (CA) group of a case series of RPL to compare the spectrum of cytogenomic abnormalities between the two groups and to evaluate the patterns of cytogenetic findings within the CA group. A skewed likelihood of recurrent normal or abnormal karyotypes was noted in consecutive miscarriages. Further genomic analysis to reveal the extent of genetic defects and functional analysis to define underlying mechanisms causing miscarriages should be considered.

## Materials and Methods

### Case Series

In this retrospective study, we retrieved karyotyping and CMA results from POC specimens with clinical indications of “RPL,” “multiple miscarriages (>2),” “multiple spontaneous abortions (×2–5),” and “fetal demise or intrauterine fetal demise” referred to the Yale Clinical Cytogenetics Laboratory during a 16-year period of 2007 to 2023. 15 A small portion of cases was also noted with suspected fetal anomalies ( *n* = 43), preterm premature rupture of the membranes (PPROM; *n* = 11), holoprosencephaly/anencephaly/acrania ( *n* = 5), cystic hygroma ( *n* = 4), and neural tube defect/spina bifida ( *n* = 3). Women of RPL were divided into two groups: an SA group with cytogenomic analysis performed once or only one result, and a CA group with cytogenomic analyses performed on consecutive miscarriages. This project was categorized as a chart review retrospective case study and deemed exempt from institutional review board (IRB) approval and granted waiver of consent based on the policy of the Yale University IRB.

### Karyotyping and Chromosome Microarray Analysis

Cell culture of chorionic villi and/or fetal skin tissue dissected from POC specimens and chromosome analysis were performed following the laboratory's standardized procedures. 16 For cases with cell culture failure or a normal karyotype, CMA was recommended and performed upon referring physician approval and insurance prior authorization. Genomic DNA was extracted from dissected POC tissues using the Gentra Puregene Kit (Qiagen, Valencia, CA). CMA was performed using the oligonucleotide array comparative genomic hybridization (aCGH, SurePrint G3 Human 8 × 60K, Agilent Inc, Santa Clara, CA) or the SNP-based OncoScan microarray assay (OMA, Affymetrix Inc. Santa Clara, CA) following manufacturers' protocols and laboratory's standardized procedures. 5 17 The aCGH data were processed through Agilent's CytoGenomics Software; the OMA data were analyzed using Affymetrix's Chromosome Analysis Suite version 3.3. The detected genomic imbalances and pCNVs were reported based on the February 2009 assembly of human genome (GRCh37/hg19).

### Statistical Analysis

The abnormalities were categorized into numerical and structural chromosomal abnormalities. The numerical chromosomal abnormalities were further divided into simple aneuploidies (common/rare autosomal trisomies and sex chromosome aneuploidies), compound aneuploidies, polyploidies (triploidy and tetraploidy), and mosaic abnormalities. The structural chromosomal abnormalities were further divided into chromosomal rearrangements and pCNVs. To compare the spectrum of abnormalities between SA and CA groups, a chi-square test was performed to evaluate the equality of proportions of abnormalities at a 95% confidence level. The Pearson chi-square test was applied to determine the likelihood of the observed distribution fitting the expected distribution in the CA group. Categorical variables were presented as a percentage of total cases (%) and a chi-square test value *p* < 0.05 was accepted as statistically significant.

## Results

**Fig. 1:**
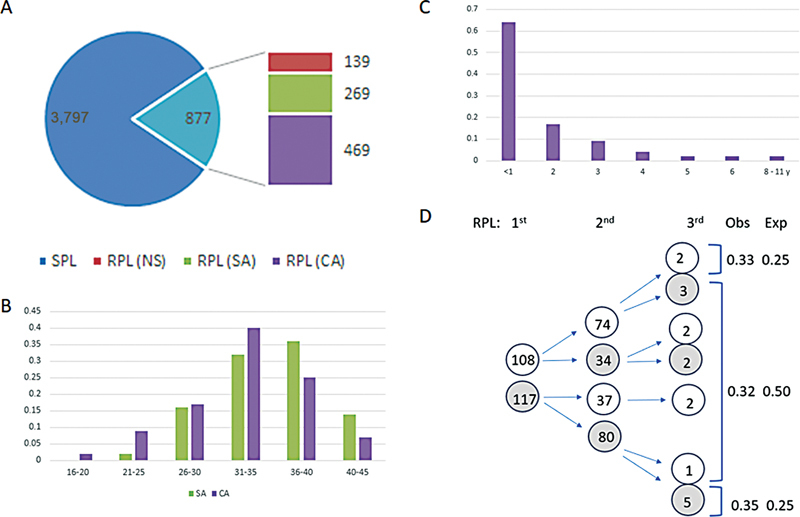
Demographics of RPL cases in the SA and CA groups. ( **A** ) Proportions of SPL and RPL cases from a total of 4,674 POC specimens. The 877 RPL cases were divided into no study (NS), single analysis (SA), and consecutive analysis (CA) groups. ( **B** ) Maternal age distribution at the time of first cytogenetic analysis. ( **C** ) Time intervals between first and second or third miscarriages from the CA group. ( **D** ) Patterns of normal ( *white circle, number inside for observed cases* ) versus abnormal ( *gray circle* ) cytogenetic findings in the CA group with observed an expected distributions from the first and second miscarriages.

Over a 16-year period of 2007 to 2023, cytogenetic analysis was performed on 4,674 POC specimens, which included 3,797 cases (81%) referred for SPL and 877 cases (19%) referred for RPL. For the 877 POC specimens by RPL, 113 specimens (13%) were not studied due to the absence of fetal tissue and the presence of only maternal decidua, 26 specimens (3%) yielded no result due to culture failure, 269 specimens (31%) from 266 women (including three twin pregnancies) with a single result from their miscarriages were collected in the SA group, and 469 specimens (53%) of consecutive miscarriages from 225 women were collected in the CA group ( Fig. 1A ). The maternal age at the time of first cytogenetic testing for women in the SA and CA groups showed a similar distribution with 2 and 9% collected at 21 to 25 years, 16 and 17% at 26 to 30 years, 32 and 40% at 31 to 35 years, and 36 and 25% at 36 to 40 years, respectively ( Fig. 1B ).

### Cytogenomic Findings in the Single Analysis Group

The results of karyotyping and CMA from the SA group are listed in Table 1 . Of these 269 POC specimens (three twin miscarriages) from 266 women, normal karyotypes were noted in 98 cases (36%) and chromosomal abnormalities were noted in 171 cases (64%). Simple aneuploidies, compound aneuploidies, polyploidies, structural abnormalities/pCNVs, and mosaic abnormalities were detected in 109 cases (41%), five cases (2%), 35 cases (13%), 10 cases (4%), and 12 cases (4%) respectively. The most frequently observed numerical abnormalities were trisomy 16 (33/269, 12%), triploidy (29/269, 11%), trisomy 22 (19/269, 7%), monosomy X (14/269, 5%), trisomy 15 (13/269, 5%), trisomy 21 (10/269, 4%), and tetraploidy (6/269, 2%). Other autosomal aneuploidies included trisomies 9 and 14 in four cases each; trisomy 4 in three cases; trisomy 12 in two cases; and trisomies 2, 3, 8, 17, and 20 and monosomy 21 in one case each. Compound aneuploidy of two to three chromosomes was seen in five cases involving chromosomes X, 4, 8, 15, 16, 20, 21, and 22. Mosaic abnormalities in 12 cases were aneuploidy or polyploidy with a normal karyotype.

**Table 1:** Cytogenomic abnormalities detected in SA and CA groups of RPL

	RPL-SA	%	RPL-CA	%	RPL a	%
Normal cases	**98**	**36%**	**227**	**48%**	**325**	**44%**
Abnormal cases	**171**	** 64% b**	**242**	** 52% b**	**413**	** 56% b**
Numerical abnormalities	**149**	** 55% b**	**207**	** 44% b**	**356**	** 48% b**
Simple aneuploidy	**109**	**41%**	**160**	**34%**	**269**	**36%**
Common autosomal trisomies	**76**	**28%**	**103**	**22%**	**179**	**24%**
47, + 16	33	12% b	27	6% b	60	8% b
47, + 22	19	7%	25	5%	44	6%
47, + 15	13	5%	14	3%	27	4%
47, + 21	10	4%	11	2%	21	3%
47, + 13	1	0	14	3%	15	2%
47, + 18	0	0	12	3%	12	2%
Rare autosomal trisomies	**19**	**7%**	**33**	**7%**	**52**	**7%**
Sex chromosomes	**14**	**5%**	**24**	**5%**	**38**	**5%**
45,X	14	5%	22	5%	36	5%
47,XXY	0	0	2	0	2	0
Compound aneuploidy	**5**	**2%**	11	**2%**	**16**	**2%**
Polyploidy	**35**	**13%**	**36**	**8%**	**71**	**10%**
Triploidy (3n)	29	11%	30	6%	59	8%
Tetraploidy (4n)	6	2%	6	1%	12	2%
Structural abnormalities	**10**	**4%**	**19**	**4%**	**29**	**4%**
Chromosomal rearrangements	7	3%	17	4%	24	3%
pCNVs	3	1%	2	<1%	5	1%
Mosaic abnormalities	**12**	**4%**	**16**	**3%**	**28**	**4%**
Total	**269**		**469**		**738**	

Abbreviations: CA, consecutive analysis; RPL, recurrent pregnancy loss; SA, single analysis.

Note: Bold signifies major categories of cytogenomic findings.

a Data from SA + CA.

b Significant difference noted between the SA and CA groups, but no difference between the CA and RPL groups.

**Fig. 2:**
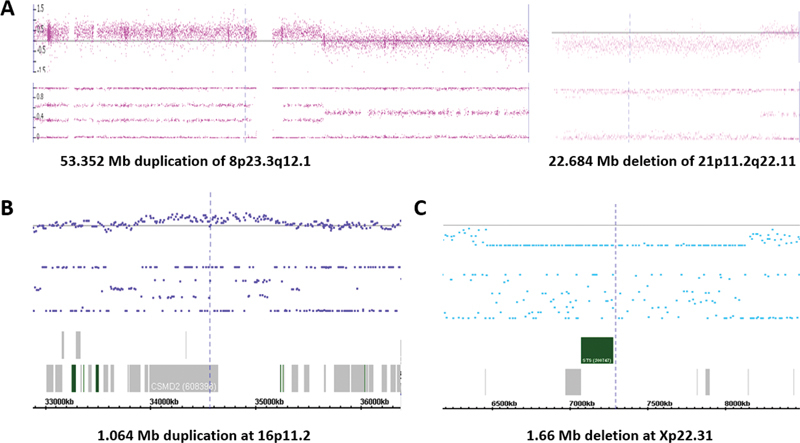
Chromosome microarray results on three POC specimens. ( **A** ) A 53.352 Mb duplication of 8p23.3q12.1 and a 22.684 Mb deletion of 21p11.2q22.11 resulted from a derivative chromosome 8. ( **B** ) A 1.064 Mb duplication at 16p11.2. ( **C** ) A 1.66 Mb deletion at Xp22.31. Top panel shows log2 ratio, and lower panel shows biallelic frequency, and bottom panel (in B/C) shows genomic coordinates and genes.

Structural chromosomal abnormalities detected in seven cases and pCNVs in three cases are shown in Table 2 . The most frequently seen recurrent structural abnormalities were Robertsonian translocations resulting in gains of 14q in three cases. One case had an extra Robertsonian translocation of 14q/14q for four copies of 14q, and two cases showed three copies of 14q from Robertsonian translocations of 14q/14q and 14q/22q. Four cases were detected with unbalanced rearrangements involving chromosomes 1q/11q, 2q, 8q/21p, and 18p/q. CMA defined a 53.352 Mb duplication of 8p23.3q12.1 and a 22.684 Mb deletion of 21p11.2q22.11 in a male complement, likely the result of a derivative chromosome from an 8q/21q translocation ( Fig. 2A ). CMA detected pCNVs in three cases. One case had a 1.064 Mb duplication at 16p11.2 which encompasses 27 OMIM genes including the *KIF22* (OMIM#603213), *PRRT2* (OMIM#614386), *TLCD3B* (OMIM#615175), *ALDOA* (OMIM#103850), *TBX6* (OMIM#602427), and *CORO1A* (OMIM#605000) morbid genes ( Fig. 2B ). This duplication overlaps with the dosage-sensitive 16p11.2 region (proximal, BP4–BP5) (includes *TBX6* ) and is diagnostic for chromosome 16p11.2 duplication syndrome (OMIM#614671). The second case had a 1.411 Mb deletion at 17q12 which encompasses *14 OMIM* genes including the *ZNHIT3* (OMIM#604500), *PIGW* (OMIM#610275), *ACACA* (OMIM# 200350), and *HNF1B* (OMIM#189907) morbid genes. This deletion includes the dosage-sensitive 17q12 region (includes *HNF1B* ) and is diagnostic for chromosome 17q12 deletion syndrome (OMIM#614527). The third case had a 7.6 Mb deletion at Xp22.33p22.31 in a male complement which encompasses 27 OMIM genes, including the *SHOX* (OMIM#312865), *CSF2RA* (OMIM#306250), *XG* (OMIM#314700), *ARSL* (OMIM#302950), *NLGN4X* (OMIM#300427), and *STS* (OMIM#300747) morbid genes. This deletion overlaps with the dosage-sensitive Xp22.31 recurrent region (includes STS), and deletion of this region is associated with X-linked recessive ichthyosis (OMIM#308100).

**Table 2:** Structural abnormalities and pCNVs detected in SA and CA groups

Case No.	Karyotype/CMA (GRCh37/hg19)
SA	
SA143	46,XY,der(1)t(1;11)(q43;q14.1)mat
SA173	46,XY,add(2)(q37.3)
SA139	46,XX,+14,rob(14;14)(q10;q10)
SA183	47,XX,+rob(14;14)(q10;q10)
SA51	46,XY,+14,rob(14;22)(q10;q10)
SA231	mos 46,XX,del(18)(p10)[11]/46,XX,i(18)(q10)[4]
SA26	arr 8p23.3q12.1(172416_56524696)x3,21p11.2q22.11(9648314_32332613)x1
SA35	46,XX.arr 16p11.2(29133476_30197490)x3
SA209	46,XX.arr 17q12(34832202_36243169)x1
SA215	arr Xp22.33p22.31(177941_7792383)x0
CA	
CA37	46,X,-X,+der(6)t(X;6)(q13;q16.2)mat
CA11	46,XX,t(3;5)(q25;q22).arr(X,1–22)x2
CA136	47,XX,t(6;9)(p21.1;q34),+14
CA149	46,XY,+der(7)t(7;15)(q31;q11.2),−15
CA149	46,XX,+der(7)t(7;15)(q31;q11.2),−15
CA166	mos 46,XY,add(8)(p11.2)[7]dn/46,XY[13]
CA128	46,XY,i(8)(q10),der(9)t(8;9)(p11;p24)
CA163	48,XX,+9,rob(14;14)(q10;q10),+15
CA213	47,XX,t(11;22)(q23;q11.2),+16
CA213	47,XX,+der(22)t(11;22)(q23;q11.2).arr 11q23.3q25(116690577_134938847)x3,22q11.1q11.21(16054712_20747763)x3
CA157	46,XY,del(13)(q13q31).arr 13q13.1q31.1(32913501_82562672)x1
CA207	46,XY,del(13)(q11)
CA190	46,XX,der(18)t(18;21)(q23;q22.2)mat
CA151	46,XY,der(20)t(11;20)(q21;p13)pat
CA196	46,XY,i(22)(q10)
CA50	47,XX,+mar.arr 22q11.1q11.21(16054712_21968221)x4
CA26	mos 46,X,r(X)[45]/45,X[4].arr Xq27.1q28(139012778–155260560)x1
CA210	arr 7q11.23(72726378_74139531)x1
CA134	46,XY.arr Xp22.31(6458165_8116400)x0

Abbreviations: CA, consecutive analysis; CMA, chromosome microarray analysis; pCNV **,** pathogenic copy number variants; SA, single analysis.

### Cytogenomic Findings in the Consecutive Analysis Group

Cytogenomic results on 469 POC specimens from consecutive miscarriages of 225 women in the CA group are listed in Table 1 . Normal karyotypes were noted in 227 cases (48%), and chromosome abnormalities were detected in 242 cases (52%). Simple aneuploidies, compound aneuploidies, polyploidies, structural rearrangements/pCNVs, and mosaic abnormalities were detected in 160 cases (34%), 11 cases (2%), 36 cases (8%), 19 cases (4%), and 16 cases (3%), respectively. The most frequently observed numerical abnormalities were triploidy (30/469, 6%), trisomy 16 (27/469, 6%), trisomy 22 (25/469, 5%), monosomy X (22/469, 5%), trisomy 13 (14/469, 3%), trisomy 15 (14/469, 3%), trisomy 18 (12/469, 3%), trisomy 21 (11/469, 2%), and tetraploidy (6/469, 1%). Other autosomal aneuploidies included trisomies 14 and 20 in five cases each, trisomy 9 in four cases, trisomies 2, 4, and 8 in three cases each, trisomies 3, 12, and 17 in two cases each, and trisomies 6, 7, 10, and 11 in one case each. Compound aneuploidy of two to three chromosomes was seen in 11 cases involving chromosomes X, Y, 8, 9, 10, 13, 14, 15, 16, 18, 20, 21, and 22. Mosaic abnormalities in 16 cases were aneuploidy or polyploidy with a normal karyotype.

Structural chromosomal abnormalities detected in 17 cases and pCNVs in two cases are shown in Table 2 . Recurrence of structural abnormalities derived from parental translocations *t* (7;15) and *t* (11;22) were noted in two women. CMA further defined the genomic imbalances of a large 49.65 Mb deletion of 13q13.1q31.1, a marker chromosome for a 5.91 Mb triplication of 22q11.1q11.21, and a 16.43 Mb deletion in a ring chromosome X. CMA detected a pCNV of a 1.41 Mb deletion at 7q11.23 with a female complement in one POC, which encompasses 25 OMIM genes including the *DNAJC30* (OMIM#618202), *ELN* (OMIM#130160), and *FKBP6* (OMIM#604839) morbid genes. This deletion overlaps with the dosage-sensitive 7q11.23 region and is diagnostic of Williams–Beuren syndrome (WBS, OMIM#194050). Another POC with a 1.66 Mb deletion at Xp22.31 in a male complement encompasses the dosage-sensitive *STS* gene (OMIM#300747) for X-linked recessive ichthyosis (OMIM#308100) ( Fig. 2C ).

### Spectrum and Patterns of Cytogenomic Findings in the Single Analysis and Consecutive Analysis Groups

The spectrum of cytogenomic abnormalities from the SA and CA groups and combined as RPL is shown in Table 1 . The abnormality detection rate of 64% in the SA group, 52% in the CA group, and 56% in RPL showed significant differences among the three groups (SA vs. CA vs RPL), between the SA and CA groups and the SA and RPL groups ( *p* < 0.05), but no difference between the CA and RPL groups ( *p* = 0.16). There were no significant differences in the detection of various types of numerical and structural abnormalities and pCNVs among the three groups ( *p* > 0.05), except for a significant difference in the detection of trisomy 16 in the above group comparisons ( *p* < 0.05) and no difference between the CA and RPL groups ( *p* = 0.12). The noted differences could be induced by the small sample size of the SA group. The lower detection rate of some specific chromosomal abnormalities such as trisomy 13, trisomy 18, and XXY in the SA group could also be explained by the small sampling size. The abnormality detection rate of 56% from 738 cases of RPL was comparable to the 49% from 1,001 POC cases of SPL in a previous study. 5 In summary, a similar spectrum of numerical and structural chromosomal abnormalities was noted in the SA and CA groups. Common autosomal trisomies of chromosomes 13, 15, 16, 18, 21, and 22, rare autosomal trisomies of other autosomes, sex chromosome aneuploidies, compound aneuploidy, and polyploidy were detected in approximately 24, 7, 5, 2, and 10% of RPL, respectively. Chromosomal rearrangements and pCNVs were detected in 3 and 1% of RPL, respectively. Mosaic abnormalities were detected in approximately 4% of RPL.

For the 225 women in the CA series, the time interval for the second or third miscarriages within 1, 2, 3, 4, and 5+ years were seen in 64, 17, 9, 4, and 6% of RPL, respectively, indicating approximately 80% of consecutive miscarriages occurred within 2 years ( Fig. 1C ). Based on the cytogenetic findings from the first and second miscarriages, patterns of recurrent normal karyotypes, alternating normal and abnormal karyotypes, and recurrent abnormal karyotypes were observed in 74 (33%), 71 (32%), and 80 (35%) of consecutive miscarriages. Thus, a skewed likelihood of 68% for recurring normal (74/108) or abnormal karyotypes (80/117) and 32% for alternating karyotypes in subsequent miscarriages was noted ( Fig. 1D ). The third miscarriages included results from only 17 POC specimens and were insufficient to evaluate this skewed likelihood but a continuous tendency was suggested. For the 80 women with abnormal karyotypes in consecutive miscarriages, 86% (69/80) had recurrent numerical abnormalities involving different chromosomes, 11% (9/80) had repeat numerical abnormalities, and approximately 3% (2/80) were caused by parental carriers of a balanced translocation ( Tables 2 ). As shown in Table 3 , repeat numerical abnormality in consecutive miscarriages included monosomy X in two women, trisomy 16 in one woman, triploidy in five women, and tetraploidy in one woman. Based on previous data that approximately 50% of pregnancy losses are caused by chromosomal abnormalities, 5 18 an expected distribution for the above patterns should be 56 (25%), 113 (50%), and 56 (25%), respectively. The observed distribution was significantly different from the expected distribution with a chi-square statistic of 16.31 and *p* -value <0.05.

**Table 3:** Repeat numerical abnormalities in the CA group

Patients/RPL	1	2	3
CA19	45,X	45,X	
CA59	45,X	45,X	
CA219	47,XY,+16	47,XX,+16	
CA35	69,XXY	68,XXY,−3	
CA93	70,XXY,+6	69,XXY	
CA119	69,XXY	69,XXY	
CA177	69,XXX	69,XXX	
CA186	69,XXY	69,XXY	
CA44	92,XXXX	92,XXXX	92,XXXX

Abbreviations: CA, consecutive analysis; RPL, recurrent pregnancy loss.

## Discussion

Current cytogenetic testing on POC is rarely performed on the first pregnancy loss due to the low risk (3–5%) of RPL and high success rate in following pregnancies. The cytogenetic results from so-called “SPL” may represent the first cytogenetic evaluation on unspecified RPL events. A retrospective analysis of 12,096 POC specimens over a 20-year period revealed similar rates and patterns of aneuploidies, sex chromosome abnormalities, triploidies, tetraploidies, complex abnormalities, and structural rearrangements between patients with a single spontaneous miscarriage and RPL. 12 A recent meta-analysis on a total of 8,320 POC in 19 studies suggested that the incidence of chromosomal abnormalities in SPL was significantly higher than that in RPL, but the distribution of types of abnormalities showed no differences between SPL and RPL. However, the prevalence and distribution of chromosomal abnormalities could be affected by the size of sampling, the year of publication, regional practice standards, variability in patient selection and inclusion, and methods of analysis. 14 Our analysis revealed a similar spectrum of cytogenomic abnormalities between the SA and CA groups of RPL, which was also comparable to that observed from previous studies on SPL and RPL. 4 5

A recent study on a large case series of 1,081 PRL specimens detected abnormal karyotypes in 36% of them, including approximately 30% of patients with two or more POC specimens analyzed; recurrent abnormalities related to parental chromosomal rearrangements, recurrent polyploidy, repeated and recurring aneuploidy were detected in 8, 7.5, and 4% of patients, respectively. 12 Our results from the CA group indicated an abnormality detection rate of 51% (114/225) in the second miscarriage and 59% (10/17) in the third miscarriage ( Fig. 1D ). Results from previous studies and this case series supported a recommendation of cytogenetic analysis on consecutive miscarriages. 11 12 13 14

Patterns of recurrent normal karyotypes, alternating normal and abnormal karyotypes, and recurrent chromosomal abnormalities were observed in 33, 32, and 35% of 225 RPL women in the CA group. A similar distribution of 37, 27, and 35% was also observed in a study of 108 primary and secondary RPL in 51 women. 11 These observations supported a skewed likelihood of 2/3 chance to have recurrent normal and abnormal patterns and 1/3 chance for an alternating karyotype in subsequent miscarriages to maintain an overall distribution of 1/3 for each pattern with chromosomal abnormality in approximately 50% of RPL. Further genomic analysis for RPL cases with recurrent normal karyotypes could lead to the identification of embryonic lethal and detrimental variants affecting early fetal development. Exome sequencing on a cohort of SPL with normal karyotypes detected pathogenic and likely pathogenic variants in 22% of cases. 19 Embryonic lethal variants in chromosomally normal pregnancy losses were detected from a case-control study and putatively detrimental variants in a panel of genes known to be associated with miscarriages were detected from euploid miscarriages. 20 21 Exome sequencing in cases of RPL identified novel pathogenic variants in several candidate genes and their functional implications were further analyzed by gene expression knockdown using targeted siRNA in cell lines and by reduced reproductive performance in variant knock-in mice. 22 23 A new enrichment approach for candidate gene detection in unexplained RPL and implantation failure was proposed by exome sequencing, detailed bioinformatic analyses, and in silico protein structural analyses. 24

RPL with recurrent aneuploidy, compound aneuploidy, and polyploidy could be caused by pathogenic variants affecting meiotic chromosome segregation. A rare occurrence of three consecutive aneuploid pregnancies of trisomy 21, trisomy 9, and trisomy 18 suggested a high risk for nondisjunction. 25 A healthy woman ascertained by 18 consecutive miscarriages with triploidy lead to the identification of candidate variants in the *PLCD4* and *OSBL5* genes with functional implications in oocyte activation and completion of meiosis II. 26 The detection of repeated chromosomal aneuploidies and pCNVs could also suggest possible gonadal mosaicism, which could be a technical challenge for a firm diagnosis. 11 27 The repeated trisomy 16, monosomy X, and triploidy by CA are the most frequently seen numerical chromosomal abnormalities in pregnancy loss, the possibility of a coincidence event cannot be ruled out.

Structural rearrangements detected in POC could be de novo events or inherited from one of the parents carrying a balanced rearrangement. The detection rate for structural abnormalities from SA and CA cases is approximately 3%. Despite recommendations for follow-up parental studies upon the detection of structural abnormalities, only 5 out of 24 cases in this series confirmed a parental carrier of a balanced translocation. Consistent with our result, cytogenetic analysis on peripheral blood specimens from 570 and 224 couples with RPL showed a detection rate of 3% (18/57) and 7% (16/224) for parental carriers of a balanced translocation and 0.3% (2/570) and 2% (4/224) for carriers of a Robertsonian translocation, respectively, which provides cytogenetic etiology of consecutive two to nine abortions in these couples. 28 29 A recent meta-analysis focused on CMA results from large cases series of POC indicated a detection rate of approximately 3% for pCNVs and 1% for genomic disorders; the risk of pregnancy loss by pCNVs of 16p11.2 duplication, 17q12 deletion, Xp22.3 deletion, and 7q11.23 deletion was estimated in the range of 21 to 50%. 18 The detection rate of 1% for pCNVs in this case series was most likely an underestimation due to the incomplete implementation of CMA on POC specimens. For mosaic abnormalities detected in POC, confined placenta mosaicism, pseudomosaicism versus true mosaicism, and maternal cell contamination should be taken into considerations. A six-attribute classification of genetic mosaicism based on the location, pattern, and disease-causing mechanisms has been recently proposed and could be applied for mosaic abnormalities in POC. 30 The introduction of genomic sequencing on POC could detect all numerical and unbalanced structural chromosomal abnormalities, pCNVs, and single gene variants and thus provide evidence for diagnostic interpretation and functional implication on genetic causes of SPL and RPL. 24 31 However, there is a limitation on the detection of balanced chromosome rearrangements such as the Robertsonian translocations in parental carriers of SPL and RPL by CMA and genomic sequencing.

## Conclusions

Cytogenomic analysis on consecutive miscarriages of RPL is crucial for delineating the patterns of chromosomal findings. Exome and genome sequencing on RPL with recurrent normal and abnormal karyotypes is recommended to provide a comprehensive understanding of pathogenic variants in genes related to cell cycle regulation, ovarian function, implantation, placentation, embryologic development, and other associated biological processes. Genetic counseling for the extent of genetic defects is needed to improve diagnostic efficacy and clinical management for couples experiencing both SPL and RPL.
